# Does noninvasive cerebellar stimulation improve the balance and walking function of patients with stroke: A meta-analysis of randomized controlled trials

**DOI:** 10.1097/MD.0000000000030302

**Published:** 2022-09-09

**Authors:** Zhi-Yuan Wu, Yi-Qiao Wang, Xiao-Peng Wen, Mei-Ying Wang, Li-Na Wang, Li-Ming Lu, Kun-Bin Li

**Affiliations:** a Department of Neurological Rehabilitation, Zhengzhou Central Hospital Affiliated to Zhengzhou University, Zhengzhou, China; b Evidence-based Medicine and Data Science Centre, Guangzhou University of Chinese Medicine, Guangzhou, China.

**Keywords:** balance dysfunction, meta-analysis, noninvasive cerebellar stimulation, stroke, walking function

## Abstract

Investigating the efficacy and safety of noninvasive cerebellar stimulation in improving the balance and walking function of patients with stroke.

**Methods:**

We searched 7 databases for randomized controlled trials (RCTs) related to noninvasive cerebellar stimulation in the treatment of stroke. The Berg Balance Scale (BBS), 6-minute walk test (6MWT), and Barthel Index (BI) were used as the outcome indexes to evaluate balance, walking and activities of daily living (ADL). The quality of the research was evaluated using the Cochrane Risk of Bias Tool. A meta-analysis was performed to evaluate the difference between the noninvasive cerebellar stimulation and control groups. Heterogeneity tests were performed to assess differences in treatment effects across noninvasive cerebellar stimulation modalities. A sensitivity analysis was performed to evaluate the robustness of the results.

**Results:**

Seven studies were included, and 5 articles (71.43%) were rated as having a low risk of bias. Among the primary outcome indicators, 4 of the 7 articles were combined into the fixed effect model (*I^2^* = 38%, *P* = .18). Compared with the control group, noninvasive cerebellar stimulation improved the BBS score, and the difference was statistically significant (mean difference [MD]: 3.00, 95% confidence interval [CI]: 1.10–5.40, *P* = .03); the sensitivity analysis showed that the statistical model was still stable after sequentially eliminating each article. Compared with the control group, noninvasive cerebellar stimulation improved the 6MWT results of patients with stroke (MD: 25.29, 95% CI: 4.86–45.73, *P* = .02). However, noninvasive cerebellar stimulation did not improve the BI (MD: 15.61, 95% CI: −7.91 to 39.13, *P* = .19). No safety problems or adverse reactions to noninvasive cerebellar stimulation were observed.

**Conclusions:**

Noninvasive cerebellar stimulation improves balance and walking function of patients with stroke, but its effect on ADL is uncertain. Due to the methodological weaknesses in the included trials, more RCTs are needed to confirm our conclusions.

## 1. Introduction

Stroke is a common central nervous system disease that is divided into hemorrhagic stroke and ischemic stroke.^[1]^ It often leads to limb motor dysfunction. Balance and walking dysfunction are 2 of the factors that clinicians must monitor. At present, the rehabilitation methods for balance and walking function include peripheral intervention and brain stimulation.^[2,3]^ Among the many noninvasive brain stimulation techniques, transcranial magnetic stimulation (TMS), transcranial direct current stimulation (TDCS), and transcranial alternating current stimulation (TACS) have been widely used in rehabilitation of stroke.^[4–7]^ Traditional stimulation methods were often applied to the frontal lobe, dorsolateral frontal lobe, and motor area to promote the recovery of cognitive, speech, emotional, and motor functions.^[8–11]^ However, the cerebellum, as the site of stimulation, is rarely studied in the rehabilitation of stroke patients. The cerebellum is the center of balance and coordination.^[12,13]^ Direct cerebellar stimulation may regulate the primary motor cortex (M1 area), supplementary motor area, cingulate cortex, and basal ganglia.^[14,15]^ These regions are linked to the cerebellum through nerve fibers.

According to many studies, noninvasive brain stimulation promotes the recovery of balance, gait, posture control, and the improvement of daily living activities after stroke.^[2,16,17]^ In recent years, the application of noninvasive cerebellar stimulation to treat many types of dysfunction in patients with stroke has also been studied, including dysphagia,^[18]^ emotional disorders,^[19]^ balance disorders,^[16]^ coordination disorders,^[20]^ walking and daily living disorders.^[16]^ And, no serious safety problems have been identified. Even now a unified conclusion on whether noninvasive cerebellar stimulation is effective at improving balance and walking function after stroke and how it affects daily living activities has not been reached. Therefore, our goal is to evaluate published studies through a meta-analysis and draw conclusions, which will provide some suggestions for the clinical practice of noninvasive cerebellar stimulation as a treatment for stroke.

## 2. Methods

Our research report follows the Preferred Reporting Items for Systematic Reviews and Meta-Analyses (PRISMA) statement.^[21]^ The protocol was registered with the International Prospective Register of Systematic Reviews (PROSPERO), CRD42021247284. We followed the predetermined criteria in terms of population, interventions, comparators, outcomes and study designs (PICOS) to identify potentially eligible studies for the systematic review and meta-analyses.

### 2.1. Literature search

For our meta-analysis, we comprehensively searched 7 Chinese and English databases, including PubMed, Web of Science, EMBASE, Wanfang, CNKI, and VIP retrieval systems. The publication date of related literature was up to October 1, 2021.

A specific strategy for combinations of keywords and free words was used for the systematic search. The main word groups or phrases include 4 parts: (1) Stroke or ischemic stroke or hemorrhagic stroke; (2) Transcranial magnetic stimulation or transcranial direct current stimulation or TDCS or transcranial alternating current stimulation or TACS or noninvasive brain stimulation or rTMS or intermittent theta burst stimulation or iTBS or continuous theta burst stimulation or cTBS or noninvasive brain stimulation or NIBS; (3) Postural balance or balance or coordination or posture control or walking; and (4) Randomized controlled trial or controlled clinical trial or randomized or placebo or randomly or trial or groups. These words or phrases were connected by “and.” Please refer to Appendix 1 (Supplemental Digital Content, http://links.lww.com/MD/H143) for a detailed description of the search procedure.

### 2.2. Types of studies

All randomized controlled trials (RCTs) related to noninvasive cerebellar stimulation therapy in the treatment of stroke were considered, regardless of blinding, publication status, and length of trial. Nonrandomized, crossover study designs and uncontrolled trials were excluded. Moreover, other studies, including conference papers, case reports, comments, animal experiments, abstracts, and reviews, were excluded. Studies that did not provide statistical data or for which we could not obtain data by contacting the corresponding authors were also not considered.

### 2.3. Types of participants

Study participants received a clear diagnosis of the first stroke (confirmed by CT or MRI). The type of stroke was not limited. The condition of the patient was stable, and the disease course was at least 2 weeks. Age, sex, ethnicity, or disease duration restrictions were not applied.

### 2.4. Types of interventions

The main intervention methods studied in the observation group were noninvasive stimulation of the cerebellum, the stimulation method may be magnetic stimulation, electrical stimulation, or sound stimulation, along with studies that mainly used these stimuli combined with other intervention methods, but studies examining trauma or surgery were excluded.

The stimulation of the control group was generally false stimulation. Alternatively, it could be a nonconventional stimulus, such as a negative, contrary, or ineffective stimulus that has been proven.

The following exclusion criteria were used: (1) other central nervous system diseases except stroke, such as multiple sclerosis, cerebellar ataxia, primary tremor, intracranial infection, and so on; (2) the site of stimulation is not the cerebellum; (3) statistical data were not published or not available by contacting the corresponding authors; and (4) animal experiments.

### 2.5. Types of study outcome measurements

Various outcome measures were used in the selected studies. The Berg balance scale (BBS), as the main index for evaluating patients with stroke presenting balance dysfunction, is widely used in clinical practice.^[2]^ The BBS divides the balance function from easy to difficult based on 14 items. Each item is scored on 5 levels ranging from 0 to 4 points. Four points indicates that the patient can complete all the inspection items, and 0 points indicates that the action cannot be completed or the patient needs much help to complete it. The lowest score was 0, and the highest score was 56.

The second was the 6-minute walk test (6MWT), which was used as the index for evaluating walking function recovery.^[22]^ The 6MWT is a method for testing cardiopulmonary function and walking function. The specific method is to walk a predetermined path with a specified distance for 6 minutes as many times as possible. Measurement results are generally reported in meters (m) to record the total walking distance. The farther the walking distance within the specified time, the better the walking function. The Barthel Index (BI) is used to evaluate the changes in activities of daily living in patients with stroke after an intervention.^[23]^ The individual BI score depends on the measurement of a series of independent behaviors. Ten items are measured with a total score of 0 to 100 points. According to the total score, the function of daily life activities was determined. The higher the score, the more independent the ability to live, while the lower the score, the greater the dependence.

BBS, as the primary outcome indicator of this study, mainly evaluates the changes in balance function of patients with stroke after cerebellar noninvasive stimulation to understand the potential balance control effect of noninvasive cerebellar stimulation. The 6MWT and BI were used as secondary outcome indicators to evaluate the changes in walking function and the effect on activities of daily living in patients with stroke who received noninvasive cerebellar stimulation therapy. Four of the 7 included studies reported clinical scores to estimate balance function. The remaining 3 studies used functional assessments that quantified the 6WMT. Two of the 7 included studies reported clinical scores to estimate functional activities of daily living.

### 2.6. Criteria for selecting studies

Two independent researchers were responsible for the literature search to minimize bias. First, 1 reviewer identified duplicate publications using EndNote X9 (Thomson ResearchSoft, Connecticut, USA), scanned titles and abstracts of the articles, and classified them into different categories according to inclusion and exclusion criteria (first scan). Then, according to predefined criteria, 2 independent reviewers read the full texts of all potentially eligible studies. The characteristics of the study (author, publication year, and country), patient information (age, sex, number of patients, and disease duration), intervention and control group (frequency and types of therapy), outcome measures, the results, and adverse events were extracted.

### 2.7. Quality assessment

The risk of bias and quality of each included article were evaluated by 2 independent researchers using the Cochrane Risk of Bias Tool.^[24]^ This tool consists of 7 domains: random sequence generation, allocation concealment, blinding method, incomplete outcome data, selective outcome reporting, and other sources of bias. Each item was classified as having a low, unclear, or high risk of bias. We defined high-quality articles when 4 or more domains had a low risk of bias. Disagreements about the risk of bias assessments were resolved by consensus or by consulting a third researcher.

### 2.8. Data extraction

Both authors extracted the following data from each article: sample size, characteristics of patients, type of active stimulation, treatment protocol, pattern of the control group, and measured outcomes. The means and standard deviations (M ± SD) of the primary and secondary outcomes were retrieved from the articles. If data were reported at different time points, those collected immediately after the intervention were used. Any discrepancies about study inclusion and data extraction were resolved by discussion. Discrepancies in interpretation were resolved by discussion and mutual agreement.

### 2.9. Statistical analysis

Standard meta-analytic methods were conducted for the outcomes of each trial to estimate an overall effect on the noninvasive cerebellar stimulation group compared with the control group after treatment using RevMan 5.3 software (the Cochrane Collaboration Information Management System, Copenhagen, Denmark). We used the mean ± standardized difference (M ± SD) to report the treatment effect, as assessment scales were available for the BBS, 6MWT, and BI. Cochran Q and Higgins and Green *I^2^* statistics were used to measure the heterogeneity of the included studies. When *P* ≥ .1 and *I^2^* ≤ 50%, the included trials were homogeneous, and the fixed effects model was used for the analysis. Conversely, *P* < .1 and *I^2^* > 50% imply that the included studies were heterogeneous, and a random effect model was used to obtain more reliable outcomes.^[24–27]^

The sensitivity analysis of the BBS was performed by sequentially excluding studies to explore the effect of each selected study on overall estimates. If the pooled effect remained unaltered after each study was removed, the results were considered relatively credible. STATA 12.0 software (StataCorp LP, College Station, TX) was used to analyze the sensitivity of only the primary outcome indicator. All *P* values were calculated using a 2-sided test, and *P* < .05 was considered statistically significant.

## 3. Results

### 3.1. Study screening

**Figure 1.**
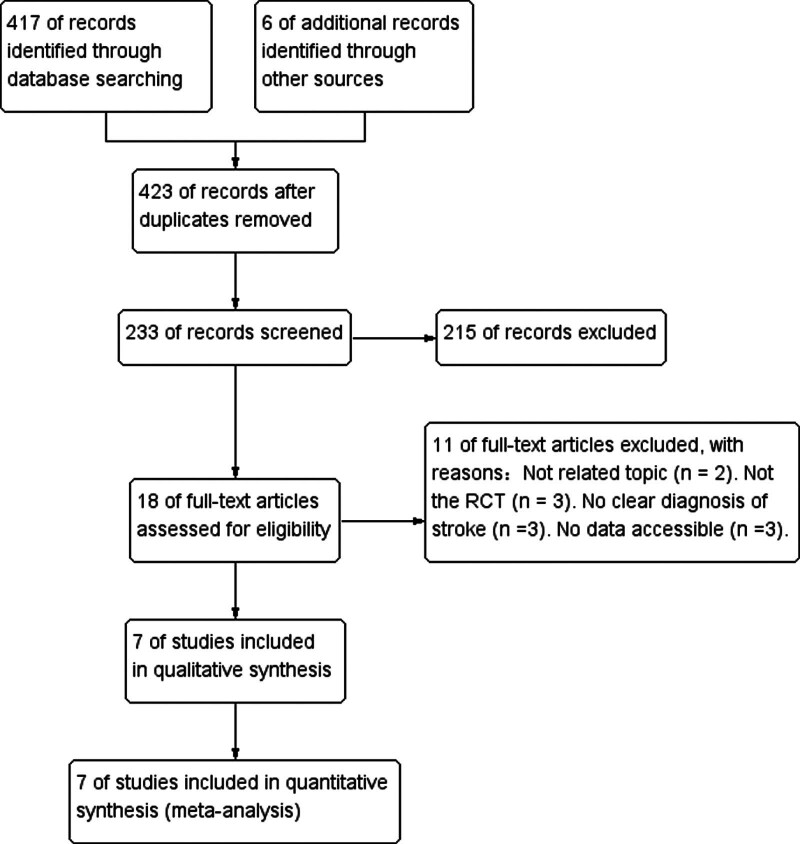
Screening process used in this study.

In Figure 1, we describe the article filtering process in detail. Initially, a computerized literature search identified 423 potentially relevant articles.

After reading the titles and abstracts, we excluded 405 articles because they were repetitive studies unrelated to our research topic, conference papers, reviews and meta-analyses, case reports, or studies of other diseases. We downloaded and read the full texts of the remaining 18 articles. Eleven articles were excluded mainly because of the following factors: (1) not related to the topic (n = 2), (2) not an RCT (n = 3), (3) no clear diagnosis of stroke (n = 3), and (4) data were not accessible (n = 3). The remaining 7 studies qualified for the meta-analysis.^[28–34]^

### 3.2. Participant and study characteristics

Seven studies qualified for this meta-analysis. These 7 studies were all randomized controlled studies. We therefore examined the therapeutic effects of noninvasive cerebellar stimulation interventions, including rTMS, TDCS and iTBS, on 212 patients with stroke. Among them, rTMS was mentioned in 3 (43%) studies, tDCS was mentioned in 3 (43%) studies, and iTBS was mentioned in 1 (14%) study. In 5 (71%) of the studies, the control groups experienced sham stimulation, while the other 2 (29%) studies used different stimulation methods. Details are shown in Table 1.

**Table 1:** Participants’ characteristics.

Outcome measure	BBS/TIS/FMA-LE/BI/Corticospinal excitability	BBS/10m-time/10m-steps	Postural sway/Wisconsin gait scale/6MWT/Time up go test	6MWT/FAC/Motricity Index/AS	6MWT/FAC/Motricity Index/AS	ICARS/BBS/BI	BBS/SI	
Treatment protocol	80% AMT, 600 stimuli, once/d, 5 d/w, 2w	1 Hz, 100% AMT, 900 stimuli, 15 min/d, 5 d/w, 1 w	1 Hz, 100% AMT, 900 stimuli, 15 min/d, 5 d/w, 4 w	2mA, 20 min/d, 5 d/w, 2w	2 mA, 20 min/d, 5/w, 2 w	1 mA, 20 min/d, the left and right cerebellums were stimulated alternately with an interval of 30 min, once/d, 5 d/w, 5 w	10 Hz; 80% AMT, 1200 pulses, 20 min/d, 5/w, 2 w
Control group	Sham	Sham	Sham	A-TDCS stimulation	Ipsilateral stimulation	Sham	Sham
Active Stimulation	iTBS	rTMS	rTMS	C-TDCS	C-TDCS	A-TDCS	rTMS
Type of stroke	Hemorrhagic/Ischemic: T (8/7), C (7/8)	Hemorrhagic/Ischemic: T (0/22), C (0/10)	Hemorrhagic/Ischemic: T 5/10), C (6/9)	Hemorrhagic/Ischemic: T (0/10), C (0/10)	Hemorrhagic/Ischemic: T (0/20), C (0/20)	Hemorrhagic/Ischemic: T (2/13), C (1/14)	Hemorrhagic/Ischemic: T (6/9), C (7/8)
Duration	T duration (70.40 ± 44.43 d)/ C duration (86.53 ± 45.26 d)	T duration (16.20 ± 13.0 d) C duration (15.10 ± 5.1 d)	T duration (75.20 ± 12.91 d) C duration (77.20 ± 10.02 d)	T duration (67.10 ± 46.75 m) C duration (51.90 ± 41.15 m)	T duration (66.4 ± 48.8 m) C duration (61.7 ± 40.1 m	T duration (66.9 ± 42.9 d) C duration (59.4 ± 61.4 d)	T duration (5.20 ± 3.60 w) C duration (5.47 ± 2.58 w)	Notes: T, treatment group; C, control group; m, male; f, female; d, day; m, month; w, week; iTBS, intermittent theta burst stimulation; rTMS stimulation; AMT, active motor threshold; BBS, Berg Balance Scale; 6MWT, 6 minute walk test
Genderr	Gender (m/f): T (12/3), C (9/6)	Gender (m/f): T (11/11), C (6/4)	Gender (m/f): T (8/7), C (7/8)	Gender (m/f): T (7/3), C (6/4)	Gender (m/f): T (10/10), C (11/9)	Gender (m/f):T (15/0), C (15/0)	Gender (m/f):T (10/5), C (9/6)	
Age	T age (51.53 ± 9.22)/C age (55.40 ± 8.10)	T age (67.4 ± 7.8)/C age (64.8 ± 11.7)	T age (61.60 ± 7.76)/C age (63.73 ± 6.10)	T age (62.6 ± 8.25)/C age (62.8 ± 11.81)	T age (63.9 ± 10.6)/C age (65.6 ± 9.7)	T age(62.7 ± 7.4)/C age (55.40 ± 8.10)	T age (55.53 ± 13.13)/C age (54.33 ± 11.46)	
Sample	n = 30	n = 34	n = 30	n = 20	n = 40	n = 30	n = 30	
Study	Liao et al.^[27]^	Kim et al.^[28]^	Cha^[29]^	Picelli et al.^[30]^	Picelli et al.^[31]^	Yuan et al.^[32]^	Zhang and Yanhua^[33]^	

Repeated transcranial magnetic stimulation.

6MWT = 6-minute walk test, A-TDCS = anodal transracial direct current, C-TDCS = cathodes transracial direct current stimulation, BBS = Berg Balance Scale, BI =Barthel Index.

### 3.3. Meta-analysis

#### 3.3.1. Berg Balance Scale.

**Figure 2.**
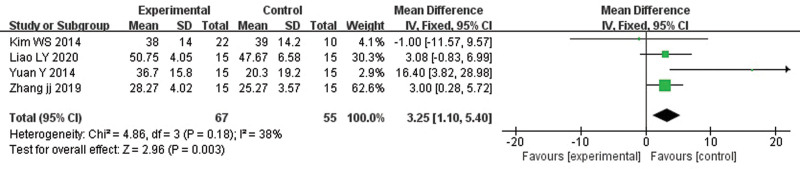
Effects of noninvasive cerebellar stimulation on the BBS. BBS = Berg Balance Scale.

Compared with the control group, noninvasive cerebellar stimulation exerted a beneficial effect on the BBS of patients with stroke (MD: 3.25, 95% CI: 1.10–5.40, *P* = .003). Heterogeneity was not present (*P* = .18). Figure 2 shows the statistical results and the forest plot.

#### 3.3.2. 6-minute walk test.

**Figure 3.**
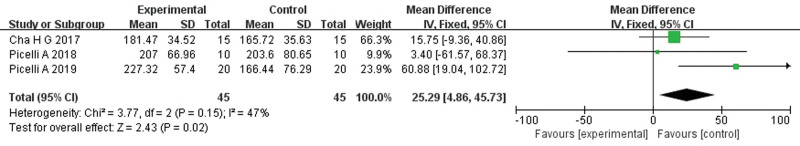
Effects of noninvasive cerebellar stimulation on the 6MWT. 6MWT = 6-minute walk test.

Compared with the control group, noninvasive cerebellar stimulation improved the 6MWT results of patients with stroke (MD: 25.29, 95% CI: 4.86–45.73, *P* = .02). Heterogeneity was not observed (*P* = .15). Figure 3 shows the statistical results and the forest plot.

#### 3.3.3. Barthel Index.

**Figure 4.**
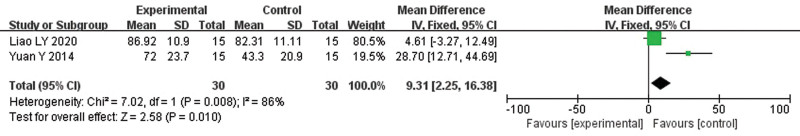
Effects of noninvasive cerebellar stimulation on BI. BI = Barthel Index.

Compared with the control group, noninvasive cerebellar stimulation improved the BI of patients with stroke (MD: 15.61, 95% CI: −7.91 to 39.13, *P* = .19). Heterogeneity was detected (*P* = .00). Figure 4 shows the statistical results and the forest plot.

### 3.4. Result of the quality assessment

**Figure 5.**
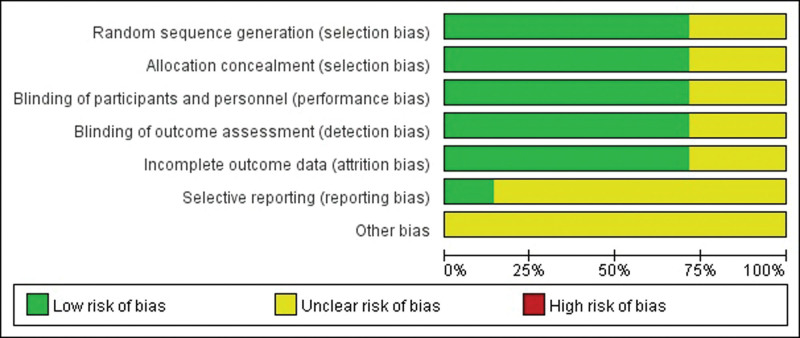
Risk of bias graph.

The Cochrane risk of bias assessment revealed some risk of bias concerns regarding selective reporting and other forms of bias. The low risk items mainly included the selection, allocation concealment, performance, detection, and attrition bias sections. The risk of bias graph is shown in Figure 5.

### 3.5. Sensitivity analysis

**Figure 6.**
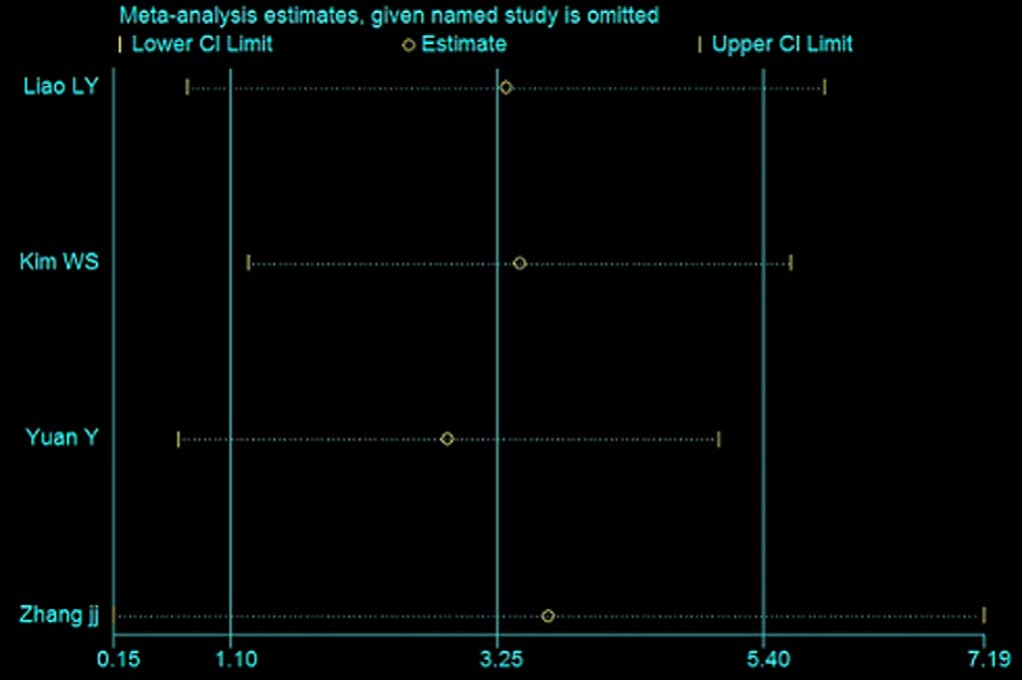
Sensitivity analysis of the primary indicator.

As shown in Figure 6, the point estimates obtained after sequentially excluding each study were consistent with the pooled effect of all studies, indicating that these data are relatively stable.

### 3.6. Adverse events and safety

Five articles discussed the safety and adverse effects of noninvasive stimulation of the cerebellum, 1 of which reported mild, nontreatable headache in subjects after treatment, but all mentioned no adverse events requiring treatment. Two articles did not mention safety or adverse reactions.

## 4. Discussion

### 4.1. Summary of results

Our meta-analysis results show that noninvasive cerebellar stimulation significantly improves the balance function and walking function of patients with stroke, but it does not clearly affect the activities of daily living of the sample. Seven articles were included in this study, among which 4 studies assessed the BBS as the primary outcome indicator, including a Chinese study published in a Chinese journal, with a total of 122 subjects. And the 6MWT and BI as secondary outcome indicators were derived from the summaries of 3 and 2 studies, respectively. Analysis results display the noninvasive cerebellar stimulation has a positive effect on the scores of BBS and BI in stroke patients, but not the ADL. Nevertheless, the total number of included studies was still low. In the literature quality assessment, reporting, and other bias have high risk bias. The reason for the analysis is that the Chinese publication had a high risk bias. In addition, the sensitivity analysis showed that the results were stable, and there were no adverse events requiring treatment in safety evaluation. Although the conclusion was positive, more rigorous RCTs are needed to support the results. Patients with stroke who were included in this study were patients with an onset time of >2 weeks, including patients with an onset time of >6 months. However, the acknowledged time required to restore brain function after stroke is 3 months.^[35]^ Because of the limited number of articles, we did not conduct a subgroup analysis stratified according to the disease course of patients.

### 4.2. Possible mechanism of noninvasive cerebellar stimulation

For motor dysfunction, stimulation of the corresponding motor area (M1 area) is generally recommended. However, the cerebellum, as a communication center, plays an important coordinating role in motor, emotional, swallowing, and cognitive functions.^[15]^ In particular, it is essential for maintaining posture, regulating muscle tension and coordinating random movement associated with posture movement.^[36]^ Therefore, an increasing number of studies have focused on improving balance and identifying relevant mechanisms through noninvasive cerebellar stimulation.

Combined with the results in which the balance and walking function of patients with stroke were affected, 3 potential mechanisms of noninvasive cerebellar stimulation have been proposed. First, cerebellar stimulation directly regulates cerebellar excitability and inhibition by stimulating different hemispheres.^[37]^ After stroke, the balance of excitation and inhibition in both cerebral hemispheres was broken. Cerebellar stimulation can promote the bilateral cerebral hemispheres to return to a balanced state again, thus promoting the recovery of dysfunction. Second, noninvasive cerebellar stimulation is involved in loop regulation of the motor learning and control.^[38]^ The circuit is the cerebellar dentate nucleus→ventrolateral nucleus of the thalamus→cortical motor area and anterior region.^[39]^ Thus, the excitability of the motor cortex was increased. Finally, specific neurons may be stimulated, which depend on the activity of aminobutyric acid and play a key role in the recovery of neurons. ^[40]^

### 4.3. Study strengths, limitations, and future directions

Our study has several advantages. First, the research method is rigorous, and the search method, covering all studies examining noninvasive cerebellar stimulation modalities, is systematic and comprehensive. Second, the cerebellum is the latest intervention site in noninvasive brain stimulation research investigated in recent years, and a new treatment direction when the development of neural rehabilitation has reached a bottleneck. Finally, as the first study assessing the effect of noninvasive cerebellar stimulation on balance function after stroke, we found that this stimulation produces positive results and is safe, suggesting that our study has good reference value for clinical rehabilitation. However it still has 2 shortcomings. Regarding the choice of articles, relatively few studies have examined noninvasive cerebellar stimulation in the rehabilitation of motor function of patients with stroke, which affects the final quality of the study. In addition, different stimulus methods were used in the included studies, and the quality of some research studies was not high. Therefore, the heterogeneity of articles must be considered.

Currently, research on noninvasive cerebellar stimulation is still in the initial stages, and many basic theoretical mechanisms remain unclear. The potential role of noninvasive cerebellar stimulation in restoring brain function and increasing the connections between the cerebral hemisphere and cortex has not been comprehensively explored.^[41]^ Some studies have shown that noninvasive cerebellar stimulation inhibits the balance function of normal humans because it inhibits the excitability of the cortical motor center.^[42]^ Some studies have reported the opposite conclusion.^[43]^ This discrepancy may be related to the use of different clinical treatment schemes, such as the stimulation site of the cerebellum (the healthy side or the affected side), the choice of stimulation frequency (low frequency or high frequency), and the level of stimulation current. Therefore, a unified conclusion on different treatment options has not been reached, which requires further research.

## 5. Conclusions

Noninvasive cerebellar stimulation improves balance and walking function of patients with stroke, but its effect on ADL is uncertain. Due to the methodological weaknesses in the included trials, more rigorous RCTs are needed to confirm our conclusions.

## Acknowledgments

Thanks are due to all authors for contributions for this paper, and we appreciate the reviewers’ valuable comments.

## Author contributions

Zhi-Yuan Wu wrote the article. Li-Ming Lu and Kun-Bin Li contributed to the conception; Yi-Qiao Wang and Xiao-Peng Wen searched the literature; Mei-Ying Wang and Li-Na Wang extracted and analyzed data. All authors have read and approved the final article.

## Supplementary Material



## References

[1] Horak FB Diener HC. Cerebellar control of postural scaling and central set in stance. J Neurophysiol. 1994;72:479–93. 7983513 10.1152/jn.1994.72.2.479

[2] Ilg W Synofzik M Brötz D. Intensive coordinative training improves motor performance in degenerative cerebellar disease. Neurology. 2009;73:1823–30. 19864636 10.1212/WNL.0b013e3181c33adf

[3] Stoodley CJ. The cerebellum and neurodevelopmental disorders. Cerebellum. 2016;15:34–7. 26298473 10.1007/s12311-015-0715-3 PMC4811332

[4] Lefaucheur JP Aleman A Baeken C. Evidence-based guidelines on the therapeutic use of repetitive transcranial magnetic stimulation (rTMS): an update (2014-2018). Clin Neurophysiol. 2020;131:474–528. 31901449 10.1016/j.clinph.2019.11.002

[5] Chung SW Hill AT Rogasch NC. Use of theta-burst stimulation in changing excitability of motor cortex: a systematic review and meta-analysis. Neurosci Biobehav Rev. 2016;63:43–64. 26850210 10.1016/j.neubiorev.2016.01.008

[6] Moura MCDS Hazime FA Marotti Aparicio LV. Effects of transcranial direct current stimulation (tDCS) on balance improvement: a systematic review and meta-analysis. Somatosens Mot Res. 2019;36:122–35. 31181963 10.1080/08990220.2019.1624517

[7] Tavakoli AV Yun K. Transcranial Alternating Current Stimulation (tACS) mechanisms and protocols. Front Cell Neurosci. 2017;11:214. 28928634 10.3389/fncel.2017.00214 PMC5591642

[8] Aleman A Enriquez-Geppert S Knegtering H. Moderate effects of noninvasive brain stimulation of the frontal cortex for improving negative symptoms in schizophrenia: Meta-analysis of controlled trials. Neurosci Biobehav Rev. 2018;89:111–8. 29471017 10.1016/j.neubiorev.2018.02.009

[9] Brunoni AR Vanderhasselt MA. Working memory improvement with non-invasive brain stimulation of the dorsolateral prefrontal cortex: a systematic review and meta-analysis. Brain Cogn. 2014;86:19. 10.1016/j.bandc.2014.01.008 24514153

[10] Wang Q Zhang D Zhao YY. Effects of high-frequency repetitive transcranial magnetic stimulation over the contralesional motor cortex on motor recovery in severe hemiplegic stroke: a randomized clinical trial. Brain Stimul. 2020;13:979–86. 32380449 10.1016/j.brs.2020.03.020

[11] Polanía R Nitsche MA Ruff CC. Studying and modifying brain function with non-invasive brain stimulation. Nat Neurosci. 2018;21:174–87. 29311747 10.1038/s41593-017-0054-4

[12] Strick PL Dum RP Fiez JA. Cerebellum and nonmotor function. Annu Rev Neurosci. 2009;32:413–34. 19555291 10.1146/annurev.neuro.31.060407.125606

[13] Amarenco P Bogousslavsky J Caplan LR. Classification of stroke subtypes. Cerebrovasc Dis. 2009;27:493–501. 19342825 10.1159/000210432

[14] Kang N Lee RD Lee JH. Functional balance and postural control improvements in patients with stroke after noninvasive brain stimulation: a meta-analysis. Arch Phys Med Rehabil. 2020;101:141–53. 31568760 10.1016/j.apmr.2019.09.003

[15] Cabanas-Valdés R Cuchi GU Bagur-Calafat C. Trunk training exercises approaches for improving trunk performance and functional sitting balance in patients with stroke: a systematic review. Neuro Rehabil. 2013;33:575–92. 10.3233/NRE-130996 24018373

[16] Koch G Bonnì S Casula EP. Effect of cerebellar stimulation on gait and balance recovery in patients with hemiparetic stroke: a randomized clinical trial. JAMA Neurol. 2019;76:170–8. 30476999 10.1001/jamaneurol.2018.3639 PMC6439971

[17] Zandvliet SB Meskers CGM Kwakkel G. Short-term effects of cerebellar tDCS on standing balance performance in patients with chronic stroke and healthy age-matched elderly. Cerebellum. 2018;17:575–89. 29797226 10.1007/s12311-018-0939-0 PMC6132826

[18] Sasegbon A Watanabe M Simons A. Cerebellar repetitive transcranial magnetic stimulation restores pharyngeal brain activity and swallowing behaviour after disruption by a cortical virtual lesion. J Physiol. 2019;597:2533–46. 30907429 10.1113/JP277545 PMC6487931

[19] Sui R Zhang L Min L. Cerebellar dysfunction may play an important role in post-stroke depression. Med Hypotheses. 2009;72:643–6. 19251375 10.1016/j.mehy.2008.11.042

[20] Bonnì S Ponzo V Caltagirone C. Cerebellar theta burst stimulation in stroke patients with ataxia. Funct Neurol. 2014;29:41–5. 25014048 PMC4172246

[21] Moher D Liberati A Tetzlaff J. Preferred reporting items for systematic reviews and meta-analyses: the PRISMA statement. . J Cli Epidemiol. 2009;62:1006–12. 10.1016/j.jclinepi.2009.06.005 19631508

[22] Kubo H Nozoe M Kanai M. Reference value of 6-minute walk distance in patients with sub-acute stroke. Top Stroke Rehabil. 2020;27:337–43. 31851872 10.1080/10749357.2019.1704372

[23] Prasad K Kumar A Misra S. Reliability and validity of telephonic Barthel Index: an experience from multi-centric randomized control study. Acta Neurol Belg. 2018;118:53–9. 29368116 10.1007/s13760-017-0843-2

[24] Higgins JP Thompson SG Deeks JJ. Measuring inconsistency in meta-analyses. BMJ. 2003;327:557–60. 12958120 10.1136/bmj.327.7414.557 PMC192859

[25] Borenstein M Higgins JP. Meta-analysis and subgroups. Prev Sci. 2013;14:134–43. 23479191 10.1007/s11121-013-0377-7

[26] Borenstein M Hedges LV Higgins JP. A basic introduction to fixed-effect and random-effects models for meta-analysis. Res Synth Methods. 2010;1:97–111. 26061376 10.1002/jrsm.12

[27] Liao LY Xie YJ Chen Y. Cerebellar theta-burst stimulation combined with physiotherapy in subacute and chronic stroke patients: a pilot randomized controlled trial. Neurorehabil Neural Repair. 2021;35:23–32. 33166213 10.1177/1545968320971735

[28] Kim WS Jung SH Oh MK. Effect of repetitive transcranial magnetic stimulation over the cerebellum on patients with ataxia after posterior circulation stroke: a pilot study. J Rehabil Med. 2014;46:418–23. 24658396 10.2340/16501977-1802

[29] Cha HG. The effect of low-frequency (1 Hz) rTMS on the cerebellar cortex in patients with ataxia after a posterior circulation stroke: randomized control trial. J Magn. 2017;22:625–9.

[30] Picelli A Chemello E Castellazzi P. Combined effects of cerebellar transcranial direct current stimulation and transcutaneous spinal direct current stimulation on robot-assisted gait training in patients with chronic brain stroke: a pilot, single blind, randomized controlled trial. Restor Neurol Neurosci. 2018;36:161–71. 29526857 10.3233/RNN-170784

[31] Picelli A Brugnera A Filippetti M. Effects of two different protocols of cerebellar transcranial direct current stimulation combined with transcutaneous spinal direct current stimulation on robot-assisted gait training in patients with chronic supratentorial stroke: A single blind, randomized controlled trial. Restor Neurol Neurosci. 2019;37:97–107. 30958319 10.3233/RNN-180895

[32] Yuan Y Dongyu W Wang J. Curative effect observation of transcranial direct current stimulation on ataxia after cerebellar stroke. Chinese J Rehabilitation Med. 2014;29:666–8.

[33] Zhang J Yanhua S. Effect of cerebellum repetitive transcranial magnetic stimulation on balance function in stroke patients. Med J Commun. 2019;33:605–6.

[34] Zeiler SR Krakauer JW. The interaction between training and plasticity in the poststroke brain. Curr Opin Neurol. 2013;26:609–16. 24136129 10.1097/WCO.0000000000000025 PMC4012223

[35] Marsden JF. Cerebellar ataxia. Handb Clin Neurol. 2018;159:261–81. 30482319 10.1016/B978-0-444-63916-5.00017-3

[36] Galea JM Jayaram G Ajagbe L. Modulation of cerebellar excitability by polarity-specific noninvasive direct current stimulation. J Neurosci. 2009;29:9115–22. 19605648 10.1523/JNEUROSCI.2184-09.2009 PMC2760225

[37] Taubert M Stein T Kreutzberg T. Remote effects of non-invasive cerebellar stimulation on error processing in motor re-learning. Brain Stimul. 2016;9:692–9. 27157059 10.1016/j.brs.2016.04.007

[38] Israely S Leisman G. Can neuromodulation techniques optimally exploit cerebello-thalamo-cortical circuit properties to enhance motor learning post-stroke? Rev Neurosci. 2019;30:821–37. 31194694 10.1515/revneuro-2019-0008

[39] Casula EP Pellicciari MC Ponzo V. Cerebellar theta burst stimulation modulates the neural activity of interconnected parietal and motor areas. Sci Rep. 2016;6:36191. 27796359 10.1038/srep36191 PMC5086958

[40] Jayaram G Tang B Pallegadda R. Modulating locomotor adaptation with cerebellar stimulation. J Neurophysiol. 2012;107:2950–7. 22378177 10.1152/jn.00645.2011 PMC3378372

[41] Foerster A Melo L Mello M. Cerebellar Transcranial Direct Current Stimulation (ctDCS) impairs balance control in healthy individuals. Cerebellum. 2017;16:872–5. 28456902 10.1007/s12311-017-0863-8

[42] Fisher KM Lai HM Baker MR. Corticospinal activation confounds cerebellar effects of posterior fossa stimuli. Clin Neurophysiol. 2009;120:2109–13. 19836995 10.1016/j.clinph.2009.08.021 PMC2852652

[43] Naro A Milardi D Cacciola A. What do we know about the influence of the cerebellum on walking ability? promising findings from transcranial alternating current stimulation. Cerebellum. 2017;16:859–67. 28456901 10.1007/s12311-017-0859-4

