# Supplementary material for: Does noninvasive cerebellar stimulation improve the balance and walking function of patients with stroke: A meta-analysis of randomized controlled trials
Source: Medicine (Baltimore). 2022 Sep 9;101(36):e30302. doi: 10.1097/MD.0000000000030302 (PMC10980459; doi:10.1097/MD.0000000000030302)
Supplement: Supplementary file 1 [file medi-101-e30302-s001.pdf]

PubMed Retrieval Type

#1 (Stroke[Mesh]) OR (Strokes[All Fields]) OR (Cerebrovascular Accident[All Fields]) OR (Cerebrovascular Accidents[All Fields]) OR (CVA[All Fields]) OR (Cerebrovascular Accident[All Fields]) OR (CVAs[All Fields]) OR (Cerebrovascular Accident[All Fields]) OR (Cerebrovascular Apoplexy[All Fields]) OR (Brain Vascular Accident[All Fields]) OR (Brain Vascular Accidents[All Fields]) OR (Cerebrovascular Stroke[All Fields]) OR (Cerebrovascular Strokes[All Fields]) OR (Apoplexy[All Fields]) OR (Cerebral Stroke[All Fields]) OR (Cerebral Strokes[All Fields]) OR (Acute Stroke[All Fields]) OR (Acute Strokes[All Fields]) OR (Acute Cerebrovascular Accident[All Fields]) OR (Acute Cerebrovascular Accidents[All Fields]) OR (ischemic stroke[All Fields]) OR (hemorrhagic stroke[All Fields]) OR (cerebrovascular disease[All Fields]) OR (lesioned hemisphere[All Fields]) OR (brain injury[All Fields]) OR (brain infarction[All Fields])

#2 (transcranial direct current stimulation[Mesh]) OR (transcranial direct current stimulat*[All Fields]) OR (transcranial DC stimulation[All Fields]) OR (transcranial DC stimulat*[All Fields]) OR (tDCS[All Fields]) OR (transcranial alternating current stimulation[All Fields]) OR (transcranial alternating current stimulat*[All Fields]) OR (tACS[All Fields]) OR (transcranial random noise stimulation[All Fields]) OR (transcranial random noise stimulat*[All Fields]) OR (tRNS[All Fields]) OR (transcranial N5 electric* current*[All Fields]) OR (transcranial N5 electric* stimulat*[All Fields]) OR (transcranial N5 brain stimulat*[All Fields]) OR (noninvasive N5 electric* current*[All Fields]) OR (non-invasive N5 electric* current*[All Fields]) OR (noninvasive N5 electric* stimulat*[All Fields]) OR (non-invasive N5 electric* stimulat*[All Fields]) OR (noninvasive N5 brain stimulat*[All Fields]) OR (non-invasive N5 brain stimulat*[All Fields]) OR (transcranial stimulat*[All Fields]) OR (transcranial current*[All Fields]) OR (noninvasive stimulat*[All Fields]) OR (noninvasive current*[All Fields]) OR (non-invasive stimulat*[All Fields]) OR (non-invasive current*[All Fields]) OR (atDCS[All Fields]) OR (a-tDCS[All Fields]) OR (ctDCS[All Fields]) OR (c-tDCS[All Fields]) OR (stDCS[All Fields]) OR (s-tDCS[All Fields]) OR (sotDCS[All Fields]) OR (so-tDCS[All Fields]) OR (HDtdcs[All Fields]) OR (transcranial magnetic stimulation[All Fields]) OR (transcranial magnetic stimulat*[All Fields]) OR (TMS[All Fields]) OR (repetitive transcranial magnetic stimulation[All Fields]) OR (repetitive transcranial magnetic stimulat*[All Fields]) OR (rTMS[All Fields]) OR (deep transcranial magnetic stimulation[All Fields]) OR (deep transcranial magnetic stimulat*[All Fields]) OR (dTMS[All Fields]) OR (theta burst stimulation[All Fields]) OR (theta burst stimulat*[All Fields]) OR (TBS[All Fields]) OR (iTBS[All Fields]) OR (cTBS[All Fields]) OR (navigated brain stimulation[All Fields]) OR (navigated brain stimulat*[All Fields]) OR (navigated transcranial magnetic brain stimulation[All Fields]) OR (navigated transcranial magnetic brain stimulat*[All Fields]) OR (theta-burst stimulation[All Fields]) OR (theta-burst stimulat*[All Fields])

#3 (Postural Balance[Mesh]) OR (Posture Equilibrium[All Fields]) OR (Posture Equilibriums[All Fields]) OR (Postural Equilibrium[All Fields]) OR (Posture Balance[All Fields]) OR (Posture Balances[All Fields]) OR (Musculoskeletal Equilibrium[All Fields]) OR (Postural Control[All Fields]) OR (Postural Controls[All Fields]) OR (Posture Control[All Fields]) OR (Posture Controls[All Fields]) OR (Balance[All Fields]) OR (postural responses[All Fields]) OR (postural

function[All Fields]) OR (postural stability[All Fields]) OR (static balance[All Fields]) OR (postural adjustments[All Fields]) OR (posturography[All Fields]) OR (stabilometry[All Fields]) OR(posture[All Fields])

#4 ('randomized controlled trial'[pt] OR 'controlled clinical trial'[pt] OR randomized[tiab] OR placebo[tiab] OR randomly[tiab] OR trial[tiab] OR groups[tiab]) AND 'humans'[MeSH Terms]

#5 #1 AND #2 AND #3 AND #4

Cochrane Retrieval Type

#1 Trials matching (Stroke) OR (Strokes) OR (Cerebrovascular Accident) OR (Cerebrovascular Accidents) OR (CVA) OR (Cerebrovascular Accident) OR (CVAs) OR (Cerebrovascular Accident) OR (Cerebrovascular Apoplexy) OR (Brain Vascular Accident) OR (Brain Vascular Accidents) OR (Cerebrovascular Stroke) OR (Cerebrovascular Strokes) OR (Apoplexy) OR (Cerebral Stroke) OR (Cerebral Strokes) OR (Acute Stroke) OR (Acute Strokes) OR (Acute Cerebrovascular Accident) OR (Acute Cerebrovascular Accidents) OR (ischemic stroke) OR (hemorrhagic stroke) OR (cerebrovascular disease) OR (lesioned hemisphere) OR (brain injury) OR (brain infarction) in Title Abstract Keyword

#2 (transcranial direct current stimulation) OR (transcranial direct current stimulat*) OR (transcranial DC stimulation) OR (transcranial DC stimulat*) OR (tDCS) OR (transcranial alternating current stimulation) OR (transcranial alternating current stimulat*) OR (tACS) OR (transcranial random noise stimulation) OR (transcranial random noise stimulat*) OR (tRNS) OR (transcranial N5 electric* current*) OR (transcranial N5 electric* stimulat*) OR (transcranial N5 brain stimulat*) OR (noninvasive N5 electric* current*) OR (non-invasive N5 electric* current*) OR (noninvasive N5 electric* stimulat*) OR (non-invasive N5 electric* stimulat*) OR (noninvasive N5 brain stimulat*) OR (non-invasive N5 brain stimulat*) OR (transcranial stimulat*) OR (transcranial current*) OR (noninvasive stimulat*) OR (noninvasive current*) OR (non-invasive stimulat*) OR (non-invasive current*) OR (atDCS) OR (a-tDCS) OR (ctDCS) OR (c-tDCS) OR (stDCS) OR (s-tDCS) OR (sotDCS) OR (so-tDCS) OR (HDtdcs) OR (transcranial magnetic stimulation) OR (transcranial magnetic stimulat*) OR (TMS) OR (repetitive transcranial magnetic stimulation) OR (repetitive transcranial magnetic stimulat*) OR (rTMS) OR (deep transcranial magnetic stimulation) OR (deep transcranial magnetic stimulat*) OR (dTMS) OR (theta burst stimulation) OR (theta burst stimulat*) OR (TBS) OR (iTBS) OR (cTBS) OR (navigated brain stimulation) OR (navigated brain stimulat*) OR (navigated transcranial magnetic brain stimulation) OR (navigated transcranial magnetic brain stimulat*) OR (theta-burst stimulation) OR (theta-burst stimulat*) in Title Abstract Keyword

#3(Postural Balance) OR (Posture Equilibrium) OR (Posture Equilibriums) OR (Postural Equilibrium) OR (Posture Balance) OR (Posture Balances) OR (Musculoskeletal Equilibrium) OR (Postural Control) OR (Postural Controls) OR (Posture Control) OR (Posture Controls) OR (Balance) OR (postural responses) OR (postural function) OR (postural stability) OR (static balance) OR (postural adjustments) OR (posturography) OR (stabilometry) OR(posture) in Title Abstract Keyword AND

#4 (Randomized clinical trial) OR (rct) OR (random*) in Title Abstract Keyword

#5 #1 AND 2# AND #3 AND #4

EMBASE Retrieval Type

#1 'stroke'/exp OR stroke OR strokes OR (cerebrovascular AND ('accidents'/exp OR accidents)) OR 'cva'/exp OR cva OR cvas OR (cerebrovascular AND ('accident'/exp OR accident)) OR (cerebrovascular AND ('apoplexy'/exp OR apoplexy)) OR (('brain'/exp OR brain) AND vascular AND ('accident'/exp OR accident)) OR (('brain'/exp OR brain) AND vascular AND ('accidents'/exp OR accidents)) OR (cerebrovascular AND ('stroke'/exp OR stroke)) OR (cerebrovascular AND strokes) OR 'apoplexy'/exp OR apoplexy OR (cerebral AND ('stroke'/exp OR stroke)) OR (cerebral AND strokes) OR (acute AND ('stroke'/exp OR stroke)) OR (acute AND strokes) OR (acute AND cerebrovascular AND ('accident'/exp OR accident)) OR (acute AND cerebrovascular AND ('accidents'/exp OR accidents)) OR (ischemic AND ('stroke'/exp OR stroke)) OR (hemorrhagic AND ('stroke'/exp OR stroke)) OR (cerebrovascular AND ('disease'/exp OR disease)) OR (lesioned AND ('hemisphere'/exp OR hemisphere)) OR (('brain'/exp OR brain) AND ('injury'/exp OR injury)) OR (('brain'/exp OR brain) AND ('infarction'/exp OR infarction))

#2 transcranial AND direct AND current AND ('stimulation'/exp OR stimulation) OR (transcranial AND direct AND current AND stimulat*) OR (transcranial AND dc AND ('stimulation'/exp OR stimulation)) OR (transcranial AND dc AND stimulat*) OR tdcs OR (transcranial AND alternating AND current AND ('stimulation'/exp OR stimulation)) OR (transcranial AND alternating AND current AND stimulat*) OR tacs OR (transcranial AND random AND ('noise'/exp OR noise) AND ('stimulation'/exp OR stimulation)) OR (transcranial AND random AND ('noise'/exp OR noise) AND stimulat*) OR trns OR (transcranial AND n5 AND electric* AND current*) OR (transcranial AND n5 AND electric* AND stimulat*) OR (transcranial AND n5 AND ('brain'/exp OR brain) AND stimulat*) OR (noninvasive AND n5 AND electric* AND current*) OR ('non invasive' AND n5 AND electric* AND current*) OR (noninvasive AND n5 AND electric* AND stimulat*) OR ('non invasive' AND n5 AND electric* AND stimulat*) OR (noninvasive AND n5 AND ('brain'/exp OR brain) AND stimulat*) OR ('non invasive' AND n5 AND ('brain'/exp OR brain) AND stimulat*) OR (transcranial AND stimulat*) OR (transcranial AND current*) OR (noninvasive AND stimulat*) OR (noninvasive AND current*) OR ('non invasive' AND stimulat*) OR ('non invasive' AND current*) OR atdcs OR 'a tdcs' OR ctdcs OR 'c tdcs' OR stdcs OR 's tdcs' OR sotdcs OR 'so tdcs' OR hdtdcs OR (transcranial AND magnetic AND ('stimulation'/exp OR stimulation)) OR (transcranial AND magnetic AND stimulat*) OR 'tms'/exp OR tms OR (repetitive AND transcranial AND magnetic AND ('stimulation'/exp OR stimulation)) OR (repetitive AND transcranial AND magnetic AND stimulat*) OR rtms OR (deep AND transcranial AND magnetic AND ('stimulation'/exp OR stimulation)) OR (deep AND transcranial AND magnetic AND stimulat*) OR dtms OR (theta AND burst AND ('stimulation'/exp OR stimulation)) OR (theta AND burst AND stimulat*) OR tbs OR itbs OR ctbs OR (navigated AND ('brain'/exp OR brain) AND ('stimulation'/exp OR stimulation)) OR (navigated AND ('brain'/exp OR brain) AND stimulat*) OR (navigated AND transcranial AND magnetic AND ('brain'/exp OR brain) AND ('stimulation'/exp OR stimulation)) OR (navigated AND transcranial AND magnetic AND ('brain'/exp OR brain) AND stimulat*) OR ('theta burst' AND ('stimulation'/exp OR stimulation))

OR ('theta burst' AND stimulat*)

#3 postural AND ('balance'/exp OR balance) OR (('posture'/exp OR posture) AND equilibrium) OR (('posture'/exp OR posture) AND equilibriums) OR (postural AND equilibrium) OR (('posture'/exp OR posture) AND ('balance'/exp OR balance)) OR (('posture'/exp OR posture) AND balances) OR (musculoskeletal AND equilibrium) OR (postural AND ('control'/exp OR control)) OR (postural AND controls) OR (('posture'/exp OR posture) AND ('control'/exp OR control)) OR (('posture'/exp OR posture) AND controls) OR 'balance'/exp OR balance OR (postural AND responses) OR (postural AND ('function'/exp OR function)) OR (postural AND ('stability'/exp OR stability)) OR (static AND ('balance'/exp OR balance)) OR (postural AND adjustments) OR 'posturography'/exp OR posturography OR 'stabilometry'/exp OR stabilometry OR 'posture'/exp OR posture

#4 'randomized controlled trial'/exp OR 'controlled clinical trial'/exp OR randomized:ab,ti OR placebo:ab,ti OR randomly:ab,ti OR trial:ab,ti OR groups:ab,ti

#5 #1 AND #2 AND #3 AND #4

Web of science Retrieval Type

(Stroke OR Strokes OR Cerebrovascular Accident OR Cerebrovascular Accidents OR CVA OR Cerebrovascular Accident OR CVAs OR Cerebrovascular Accident OR Cerebrovascular Apoplexy OR Brain Vascular Accident OR Brain Vascular Accidents OR Cerebrovascular Stroke OR Cerebrovascular Strokes OR Apoplexy OR Cerebral Stroke OR Cerebral Strokes OR Acute Stroke OR Acute Strokes OR Acute Cerebrovascular Accident OR Acute Cerebrovascular Accidents OR ischemic stroke OR hemorrhagic stroke OR cerebrovascular disease OR lesioned hemisphere OR brain injury OR brain infarction) AND TOPIC: (Postural Balance OR Posture Equilibrium OR Posture Equilibriums OR Postural Equilibrium OR Posture Balance OR Posture Balances OR Musculoskeletal Equilibrium OR Postural Control OR Postural Controls OR Posture Control OR Posture Controls OR Balance OR postural responses OR postural function OR postural stability OR static balance OR postural adjustments OR posturography OR stabilometry OR posture) AND TOPIC: (transcranial direct current stimulation OR tdcs OR transcranial magnetic stimulation OR tms OR NIBS or non invasive brain stimulation OR brain stimulation OR tpcs OR tacs OR transcranial pulsed current stimulation OR transcranial alternating current stimulation OR random noise)

CNKI 检索式

SU=('脑卒中'+'脑梗死'+'缺血性脑梗死'+'脑出血'+'出血性脑梗死'+'中风'+'脑血管意外'+'脑损伤')

and SU=('重复经颅磁刺激'+'经颅磁刺激'+'经颅电刺激'+'经颅直流电刺激'+'经颅交流电刺激'+'经颅随机噪声刺激'+'间歇性θ短阵脉冲刺激'+'持续性θ短阵脉冲刺激'+'θ短阵快速脉冲经颅磁刺激'+'θ脉冲刺激'+'脑无创刺激'+'小脑无创刺激')

and SU=('平衡功能'+'平衡障碍'+'姿势控制'+'协调功能'+'协调障碍'+'共济失调'+'步

行功能'+'运动障碍'+'运动功能'+'步行能力'+'平衡协调'+'下肢功能')

and SU=('随机对照'+'随机对照试验'+'病例对照研究'+'临床观察'+'临床评估'+'临床试验'+'临床效果'+'临床研究'+'疗效'+'疗效评价'+'前瞻性'+'随访'+'对比研究'+'多中心'+'随机'+'随机分配'+'对照'+'盲法'+'对照组'+'双盲法'+'单盲法'+'病例报告'+'病例研究'+'病例分析'+'病例报道')

（发表时间：between （模糊匹配），专辑导航，全部数据库：文献，跨库检索）

万方检索式

主题=("脑卒中"+"脑梗死"+"缺血性脑梗死"+"脑出血"+"出血性脑梗死"+"中风"+"脑血管意外"+"脑损伤")

and 主题=("重复经颅磁刺激"+"经颅磁刺激"+"经颅电刺激"+"经颅直流电刺激"+"经颅交流电刺激"+"经颅随机噪声刺激"+"间歇性θ短阵脉冲刺激"+"持续性θ短阵脉冲刺激"+"θ短阵快速脉冲经颅磁刺激"+"θ脉冲刺激"+"脑无创刺激"+"小脑无创刺激")

and 主题=("平衡功能"+"平衡障碍"+"姿势控制"+"协调功能"+"协调障碍"+"共济失调"+"步行功能"+"运动障碍"+"运动功能"+"步行能力"+"平衡协调"+"下肢功能")

and 主题=("随机对照"+"随机对照试验"+"病例对照研究"+"临床观察"+"临床试验"+"治疗结果"+"临床研究"+"前瞻性研究"+"对比研究"+"随机分配"+"盲法"+"对照组"+"病例报告"+"病例研究"+"病例分析")

VIP 检索式：

M=(脑卒中+脑梗死+缺血性脑梗死+脑出血+出血性脑梗死+中风+脑血管意外+脑损伤)

*(M=重复经颅磁刺激+经颅磁刺激+经颅电刺激+经颅直流电刺激+经颅交流电刺激+经颅随机噪声刺激+间歇性θ短阵脉冲刺激+持续性θ短阵脉冲刺激+θ短阵快速脉冲经颅磁刺激+θ脉冲刺激+脑无创刺激+小脑无创刺激)

*(M=平衡功能+平衡障碍+姿势控制+协调功能+协调障碍+共济失调+步行功能+运动障碍+运动功能+步行能力+平衡协调+下肢功能)

*(M=随机对照+随机对照试验 +病例对照研究+临床观察+临床评估+临床试验+临床效果+临床研究+疗效+疗效评价+前瞻性+随访+对比研究+多中心+随机+随机分配+对照+盲法+对照组+双盲法+单盲法+病例报告+病例研究+病例分析+病例报道)
